# Neural mechanisms of video learning influenced by pedagogical agents’ image and voice: an fNIRS study

**DOI:** 10.3389/fpsyg.2026.1863163

**Published:** 2026-07-10

**Authors:** Yan Ma, Luo Wang, Xiaohong Lan, Yumeng Xiao

**Affiliations:** Wisdom Education Research Institute, Chongqing Normal University, Chongqing, China

**Keywords:** fNIRS, learning effectiveness, multimedia learning, pedagogical agent, video learning

## Abstract

**Background:**

In multimedia learning environments, pedagogical agents are believed to facilitate learning through social cues. However, further research is needed to determine how to design pedagogical agents to optimize the learning experience and enhance learning outcomes.

**Aims:**

This study aims to investigate how the visual and auditory characteristics of pedagogical agents in multimedia learning influence learners’ performance and brain activation patterns during the learning process.

**Methods:**

A total of 30 participants were recruited to participate in a multimedia course on aerospace-related knowledge. The experiment employed a 2 (image type: human / animation) × 2 (voice type: human / machine) within-subjects design. Functional near-infrared spectroscopy (fNIRS) was used to measure cortical activity during the learning process, and then participants completed learning outcome tests, including knowledge retention and transfer tests.

**Results:**

Regarding learning performance, the retention test revealed a significant interaction between visual and auditory modalities, with the combination of animated agents and human voices yielding the best results; the transfer test showed a significant main effect of voice type, with human voices significantly outperforming machine voices. fNIRS results indicated that, compared to human agents, animated agents elicited higher levels of brain activation in regions such as the inferior frontal gyrus, middle temporal gyrus, frontal pole, and dorsolateral prefrontal cortex; compared to machine voices, human voices elicited stronger activation responses in regions such as the frontal pole, middle temporal gyrus, and dorsolateral prefrontal cortex. The combination of animated agents and human voices exhibited the strongest brain activation pattern, suggesting that multimodal social cues may promote higher levels of cross-modal integration processing under these conditions.

**Conclusion:**

This study reveals the mechanisms underlying the synergistic effects of a pedagogical agent’s image and voice at the behavioral and cognitive-neural levels, providing empirical evidence for the optimized design of pedagogical agents in multimedia learning environments.

## 1 Introduction

With the rapid development of multimedia technology, video learning has become an important method in modern education. Multimedia learning not only provides rich informational content but also conveys knowledge through dual channels of verbal and visual information, breaking through the temporal and spatial constraints of traditional classrooms, stimulating learners’ interest, and enhancing their motivation to learn (Mayer, 2020). However, compared to traditional classrooms, multimedia learning environments lack sufficient social interaction, which can easily lead to distractions and insufficient engagement among learners, thereby affecting learning outcomes (Fiorella and Mayer, 2022). As a key application of artificial intelligence in education, the design of pedagogical agents—specifically how they can optimize the learning experience and enhance learning outcomes—has become a frontier topic in educational psychology research.

Pedagogical agent is a visual representation displayed on a computer screen in online learning to guide and facilitate learners’ learning; it can be a real person or a virtual character (Kim, 2015; Li et al., 2025). By providing learners with socially perceptive learning experiences, they help maintain learner engagement, support cognitive processing, and enhance deep learning outcomes and interest (Veletsianos, 2010). According to social agency theory, instructional agents serve as key social cues that can simulate authentic instructional interactions through language, imagery, and emotional expression, thereby establishing an emotional connection with learners and influencing their attention, motivation, and learning outcomes (Heng et al., 2025; Mayer and DaPra, 2012). Building on this, the Cognitive-Affective-Social Theory of Multimedia Learning (CASTLE) further emphasizes that social cues in learning activate social schemas in learners’ long-term memory and influence both cognitive processes (such as selection, organization, and integration) and affective processes (motivation, emotion, and metacognition) (Schneider et al., 2022; Xiao et al., 2025). However, social agency theory suggests that poorly designed instructional agents may become “seductive details” unrelated to learning objectives, increasing learners’ external cognitive load and negatively impacting learning performance (Tao et al., 2022). Therefore, the design characteristics of pedagogical agents—particularly how their image and voice elements can facilitate rather than interfere with learning—have become a core issue in this field that urgently needs to be addressed.

Although research on pedagogical agents in the field of education is growing, the underlying mechanisms through which they influence the learning process remain to be systematically investigated. Existing studies largely rely on behavioral measures or eye-tracking technology, focusing on examining external manifestations of learning or attentional allocation (Li et al., 2023), while the mechanisms by which the characteristics of pedagogical agents influence learners’ internal cognitive and neural processes remain unclear. With advances in learning science and intelligent technologies, neuroscience has emerged as a key perspective for understanding learner motivation and cognition (Luria et al., 2021). Functional Near-Infrared Spectroscopy (fNIRS), as an emerging neuroimaging technique, offers advantages such as high ecological validity and resistance to motion artifacts, making it suitable for monitoring brain neural activity in real-world learning contexts (Scholkmann et al., 2013). By monitoring changes in the concentrations of oxygenated hemoglobin (HbO) and deoxygenated hemoglobin (HbR) in the cerebral cortex, it reflects the activation levels and functional changes in corresponding brain regions (Pinti et al., 2020). The non-invasive and non-restrictive nature of near-infrared spectroscopy allows participants to complete data collection in a near-natural reading state, offering higher ecological validity and greater practical feasibility (Ma et al., 2025). Therefore, the use of fNIRS technology can reveal the cognitive processing mechanisms of learners under different audiovisual combinations, providing direct neurophysiological evidence to test the predictions of social agency theory and cognitive load theory.

### 1.1 The impact of the pedagogical agent’s image on learning

The image of a pedagogical agent is typically conceptualized as its visual appearance, referring to the external characteristics that are directly perceivable by learners. This visual representation functions as a set of social and non-verbal cues that can influence learners’ perceptions, expectations, and related judgments of the agent (Gulz and Haake, 2006; Veletsianos, 2010; Baylor and Kim, 2005). Researchers have offered explanations from various theoretical perspectives regarding how the image of pedagogical agents affects learning. Parasocial interaction theory (PSI) posits that when students watch instructional videos, they exhibit cognitive, emotional, and behavioral responses to the image of a real teacher; higher levels of PSI are often associated with better academic performance (Beege et al., 2019). However, the Uncanny Valley effect suggests that as the realism of on-screen teachers increases, learners may experience aversion or discomfort, which can negatively impact learning (Rokeman et al., 2020).

Consequently, how the design of teaching avatars influences learning has long been a focus of research; however, empirical studies directly comparing human with animated agents remain relatively scarce. The results of existing comparative studies are not entirely consistent. Some studies have found no significant difference in learning outcomes between animated agents and human (Horovitz and Mayer, 2021; Tao et al., 2022). Polat et al. (2025) also found no significant differences in learners’ academic performance, perceived classroom engagement or social presence when watching videos featuring live instructors versus anthropomorphic animated characters. However, other studies have reached different conclusions. Wang et al. (2018) had students learn about synaptic transmission through multimedia courses featuring either embodied cartoon instructors or screen-less instructors. The results showed that cartoon instructors helped students focus more intently on the content and achieve better learning outcomes. A meta-analysis by Zhou and Yang (2023) also noted that the effectiveness of different types of image, with animated agents demonstrating a more significant advantage in promoting learning. And Castro-Alonso et al. (2021) further indicated that in multimedia instructional settings, animation or simple-looking two-dimensional agents are more effective than visually complex three-dimensional agents. Overly enhancing the realism of live-action footage can actually lead to increased learner gaze but reduced learning effectiveness (Li et al., 2024). This inconsistency may stem from the fact that most studies have compared only a single type of image, without controlling for other characteristics such as voice.

In summary, although existing research provides preliminary evidence, most studies remain primarily at the behavioral level, making it difficult to accurately understand how the image of pedagogical agents specifically influences the learning process. Therefore, this study employed multiple evaluation methods: combining fNIRS data to reveal differences in the learning process and utilizing post-test scores to analyze differences in learning outcomes, thereby comparing the effects of human agents and animated agents.

### 1.2 The impact of the pedagogical agent’s voice on learning

In multimedia learning research, the Cognitive Theory of Multimedia Learning (CTML) posits that learners select, organize, and integrate information through both visual and auditory channels, thereby forming mental representations and constructing meaning (Mayer, 2020). Within this theoretical framework, the type of voice used by instructional agents emerges as one of the key design elements influencing the learning process and learning outcomes. Existing research typically categorizes the voices of pedagogical agents into two types: human-recorded voices and machine-synthesized voices. According to the voice effect in multimedia learning, learning outcomes are better when multimedia content is narrated by a human voice than when it is narrated by a machine-synthesized voice (Atkinson et al., 2005). Because human voices possess richer intonation, emotion, and natural rhythm, they are considered better at simulating the interaction of a human teacher and are more effective at eliciting social responses from learners, thereby enhancing cognitive engagement and improving learning outcomes. For example, Liew et al. (2023), using a programming algorithms course as their learning material, found that human voices were more effective in promoting learning outcomes than machine-generated voices. Jing et al. (2024) compared the effectiveness of cute-sounding and formal computer-synthesized voices, as well as human-recorded voices, in video learning and found that human voices significantly improved learners’ attention and performance. However, some studies have reached different conclusions. Chiou et al. (2020) compared low-quality and high-quality computer-generated voices with human voices, and the results showed that although learners’ trust in and perception of human voice teaching agents were superior to those of synthetic speech, voice quality had no significant impact on learning outcomes. Craig and Schroeder (2017) found that modern computer-synthesized voices yielded better learning outcomes than classic computer-synthesized voices and human voices. Although existing research has revealed the varying effects of voice types on learning, the relative impact of human voices versus synthetic voices remains controversial. Therefore, it is necessary to further explore the effects of different pedagogical agent voice types on the learning process and learning outcomes, as well as their underlying mechanisms.

### 1.3 fNIRS and its applications in video learning

With advances in cognitive neuroscience and non-invasive neuroimaging methods, researchers have increasingly focused on the relationship between cognitive development and changes in brain function (Morita et al., 2016). Due to its non-invasive, high-resolution, and cost-effective nature, fNIRS has seen increasing application in multimedia and video education research in recent years (Sajinčič et al., 2021; Zhan et al., 2023; Ozel et al., 2023), offering a new research method for elucidating the cognitive and neural mechanisms underlying real-world learning contexts.

In video-based learning environments, fNIRS is widely used to investigate processes such as cognitive load, attentional allocation, and the processing of social cues (Zhan et al., 2023). Research indicates that higher working memory load and cognitive processing often elicit significant activation in the prefrontal cortex (Csipo et al., 2021), while cognitive processing (such as the retention, manipulation, and integration of information) significantly increases activation in the dorsolateral prefrontal cortex and the frontal pole (Kim et al., 2015); The temporal lobe regions are primarily involved in language functions and auditory perception (Benavides-Varela and Gervain, 2017; Homae et al., 2014). Tian et al. (2021) used fNIRS to find that social cues in instructional videos (such as gestures and eye contact) can promote activation in the prefrontal and temporo-parietal regions. Ma et al. (2025) found that when learning materials were presented in a spatially close configuration, activation levels in the middle temporal gyrus, frontal pole, and dorsolateral prefrontal cortex were significantly higher than in a spatially separated configuration, and learning outcomes were better. Furthermore, Johannessen et al. (2026) confirmed that fNIRS technology can achieve high-precision classification of cognitive load in both 3D virtual reality and 2D learning environments. Additionally, Li et al. (2022) used fNIRS to find that animated agents, compared to conditions without agents, enhance activation in social brain regions such as the frontal, temporal, and parietal lobes, and that the level of activation is positively correlated with learning outcomes. However, existing fNIRS studies have largely focused on single factors, with insufficient attention paid to the synergistic effects of external design elements of teaching agents (such as appearance and voice) and their underlying neural mechanisms.

In summary, fNIRS technology offers unique advantages for research on video-based learning and pedagogical agents. By monitoring brain activation patterns in learners under various audiovisual conditions, fNIRS provides important neurophysiological evidence for understanding the collaborative cognitive mechanisms underlying the processing of social cues.

### 1.4 Research questions and hypotheses

According to social agency theory, pedagogical agents in multimedia learning environments can elicit social responses from learners through social cues such as images, voices, and gestures, leading learners to perceive human-computer interaction as a social interaction process, thereby promoting deep cognitive processing and improving learning outcomes (Schneider et al., 2022; Mayer and DaPra, 2012). The Cognitive Theory of Multimedia Learning (CTML) posits that learners’ information processing is constrained by limited cognitive resources. While the synergy between visual and auditory channels can optimize the use of working memory, inappropriate external presentation formats may also increase irrelevant cognitive load, interfering with the selection, organization, and integration of information (Mayer, 2020; Li et al., 2024). Therefore, from an integrative perspective, the social cues provided by pedagogical agents may, on the one hand, promote learning by enhancing social presence, while on the other hand, they may inhibit learning outcomes by increasing additional processing burdens.

Within this framework, the type of pedagogical agent (human /animation) may influence learning through attention allocation and cognitive load pathways. Human agents possess a higher degree of realism; their rich social details tend to prioritize the learner’s attention, consuming limited cognitive resources and increasing external cognitive load, thereby diverting the learner’s focus from the learning content (Mayer, 2022); In contrast, animated agents have more simplified visual features, reducing competition from stimuli unrelated to learning and helping to focus cognitive resources on key information. According to CTML and interference theory, animated agents with lower realism may be more conducive to learning (Li et al., 2024; Castro-Alonso et al., 2021). In contrast, the “voice effect” in multimedia learning has received relatively consistent empirical support. Existing research indicates that human voices, compared to machine voices, possess greater naturalness and social orientation, making them more likely to elicit social responses from learners, thereby promoting information processing and learning transfer (Mayer and Fiorella, 2021; Xiao et al., 2025).

Furthermore, CTML emphasizes the synergistic integration of visual and auditory channels in information processing, while social agent theory suggests that multiple social cues can interact to influence learners’ social cognitive processing. This implies that the visual and auditory aspects of pedagogical agents do not function independently but may jointly influence the learning process and outcomes through multimodal integration. However, existing research has largely focused on single-modality effects, with a lack of systematic exploration of the synergistic mechanisms underlying different audiovisual combinations. To explore these issues in depth, this study combines behavioral testing with fNIRS to reveal the mechanisms underlying the influence of pedagogical agents’ audiovisual characteristics at the level of neural activation.

Based on the above theoretical analysis and the current state of research, this study proposes the following research questions and hypotheses:

Research Question 1: Do the image and voice characteristics of pedagogical agents influence learners’ learning outcomes?

Hypothesis 1: Compared to human agents, animated agents are more effective in promoting learners’ learning outcomes.

Hypothesis 2: Compared to machine voices, human voices are more effective in promoting learners’ learning outcomes.

Hypothesis 3: The combination of animated agents and human voices outperforms the other three combinations in improving learners’ learning outcomes.

Research Question 2: Do the image and voice characteristics of pedagogical agents influence brain activation patterns?

Hypothesis 4: Compared to human agents, animated agents will induce stronger brain activation in the temporo-parietal junction and prefrontal cortex, regions associated with social cognition and attentional control.

Hypothesis 5: Compared to machine voices, human voices will elicit stronger brain activation in the orbitofrontal cortex and superior temporal gyrus, regions associated with the processing of emotional and social cues.

Hypothesis 6: The combination of animated agents and human voices will elicit the highest levels of activation in key brain regions (such as the frontotemporal network).

Research Question 3: Is there a relationship between learning performance and brain activation?

Hypothesis 7: There is a correlation between activation in key brain regions and learning performance, and this relationship is influenced by the audiovisual combination.

## 2 Method

### 2.1 Participants

*A priori* sample size estimation was conducted using G*Power 3.1 software. Based on effect sizes reported in previous fNIRS studies (Ma et al., 2025; Zhang et al., 2025), an effect size of *f* = 0.25, a significance level of *α* = 0.05, and a statistical power of 0.80 were set. In the statistical model, a repeated-measures ANOVA (with within-subjects factors) was selected primarily to estimate the main effects of image type and sound type, as well as their interaction effects; the calculation indicated that the minimum required sample size was 24 participants. To account for potential data loss, the study actually recruited 35 current students (13 males and 22 females) from a university through the institution’s internal recruitment platform, aged between 20 and 26 (*M* = 23.63, SD = 1.54), all of whom had a habit of learning via video. All participants had a history of watching educational videos, came from various academic backgrounds such as education and computer science, were right-handed, had normal vision or corrected vision, were native Chinese speakers, had no history of psychiatric or neurological disorders, and had no prior experience with similar experiments. This study strictly adhered to the ethical research guidelines of the affiliated institution and has received formal approval from the institution’s Institutional Review Board. All participants signed informed consent forms prior to the experiment and received appropriate compensation upon its completion.

To ensure data quality, data were screened according to the following criteria: (1) 1 participant with extremely low total test scores (<20 points) were excluded to eliminate data points that might result from carelessness, inattention, or failure to complete the task diligently, thereby enhancing the validity and reliability of subsequent statistical analyses (Curran, 2016); (2) Excluded 3 participants whose fNIRS data contained excessive artifacts due to excessive head movement; (3) Excluded 1 participant flagged for having too many channels with poor signal quality. Ultimately, data from 30 participants (11 males and 19 females) were included in the statistical analysis.

### 2.2 Experimental design

**Figure 1:**
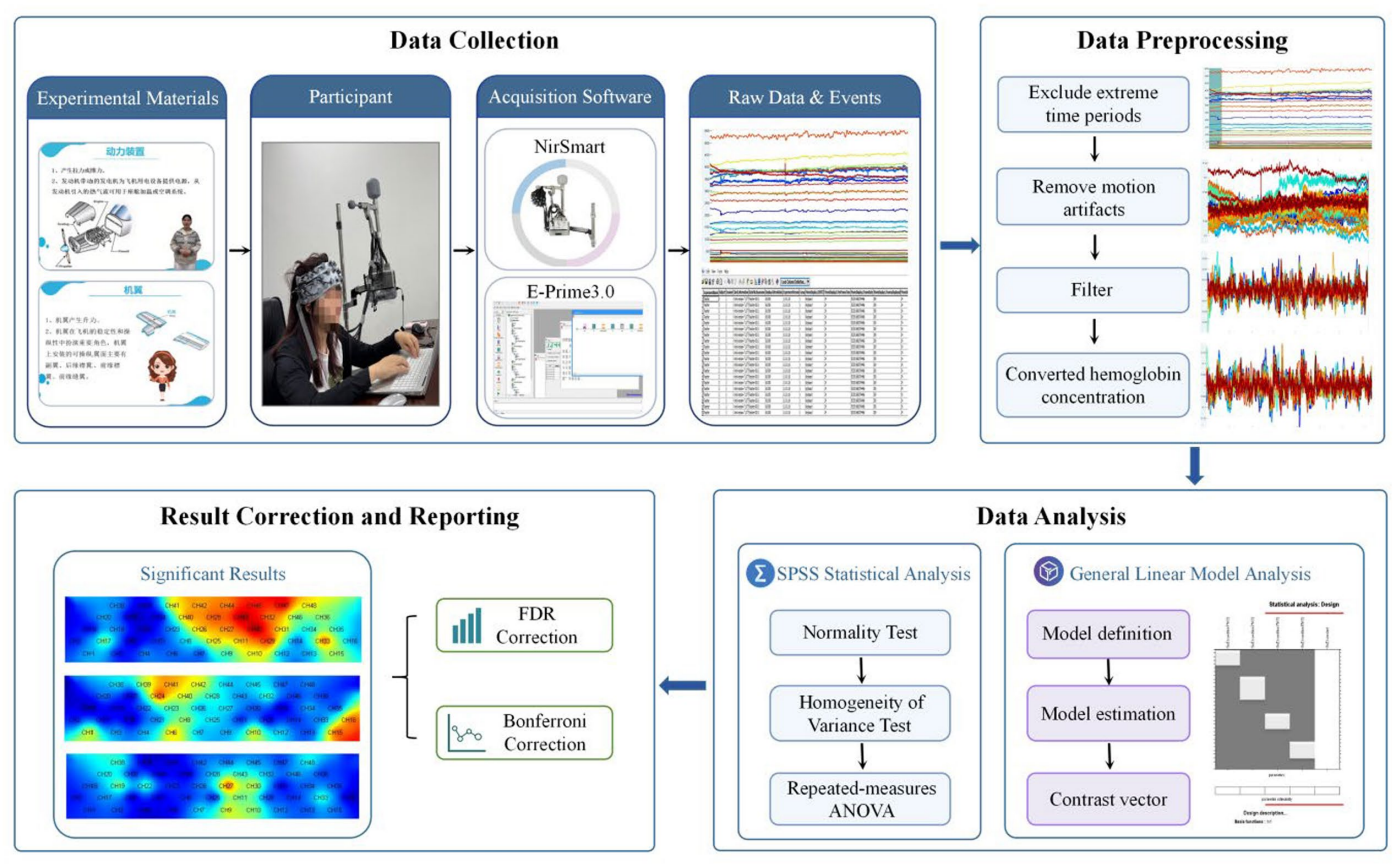
Research design framework.

The experiment employed a 2 (image type: human / animated) × 2 (voice type: human / machine) two-factor within-subjects experimental design, each participant must complete all four experimental conditions. The behavioral dependent variables were participants’ learning performance accuracy (ACC) and behavioral response time (RT) on retention and transfer tests. The neural dependent variable was the relative change in HbO during the learning phase (Castro-Alonso et al., 2021). The core framework of the experimental design is shown in Figure 1.

### 2.3 Experimental material

#### 2.3.1 Learning materials

The video learning materials were selected from the online course Understanding Flight on the Chinese University MOOC platform. The content covers the principles of aircraft flight, basic flight controls, and flight safety. This course was selected based on clear methodological considerations. First, it is designed for learners with multidisciplinary backgrounds and is of moderate difficulty, which helps minimize the influence of prior knowledge differences on learning outcomes (Kalyuga, 2007; Sweller et al., 2011). Second, the course features a clear structure and coherent organization, which facilitates maintaining consistency in the content framework across different experimental conditions, thereby controlling for potential confounding variables introduced by differences in material structure. In addition, multiple independent knowledge topics with no prerequisite dependencies were selected from this course to ensure content equivalence across conditions and to minimize cross-condition transfer and interference effects. This approach is consistent with prior multimedia learning studies that have improved internal validity by using a single course source and selecting independent knowledge topics (e.g., Sondermann and Merkt, 2023).

**Figure 2:**
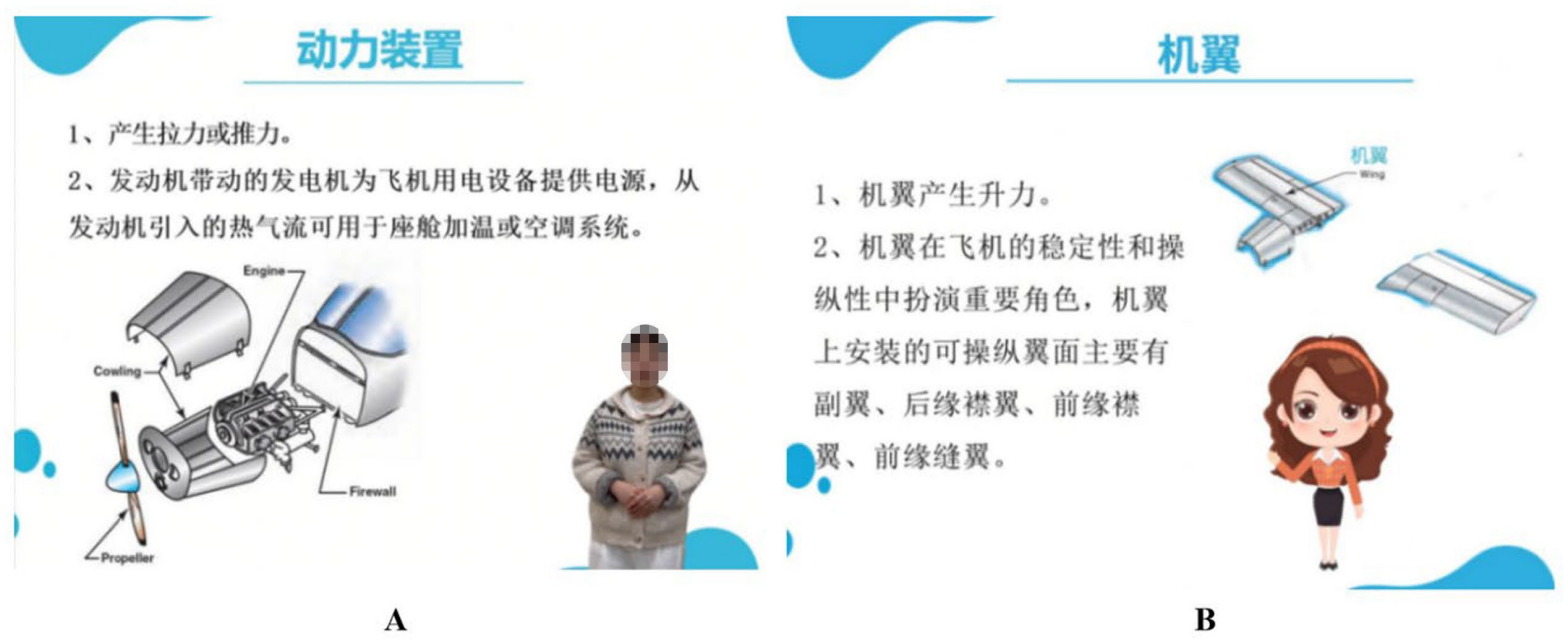
Examples of course video materials: **(A)** Human agent. **(B)** Animated agent.

We created four versions of the video based on four different learning concepts, manipulating the two dimensions of the pedagogical agent’s image and voice: human agent with human voice, human agent with machine voice, animated agent with human voice, and animated agent with machine voice. The course video materials are shown in Figure 2. The four videos correspond to four distinct, independent topics, and each topic does not rely on prior knowledge of the others, thereby minimizing transfer effects and interference effects between the content (Charness et al., 2012). The pedagogical agents were designed as follows: For the image design, the human agent was a 22-year-old female teacher with a teaching qualification certificate who appeared on screen to deliver the instruction. The animated agent was created using the preset “educational female character” model in the software Wancai Animation Master, with its size, position, and background kept consistent with those of the human agent condition. The character features a neutral and simple cartoon style, avoiding exaggerated or redundant visual details. This design is aligned with the Cognitive Load Theory in multimedia learning, which emphasizes the reduction of extraneous visual information (Mayer, 2020). Additionally, this character represents a typical educational agent model within the software. For the voice design, the human voice recordings were performed by a female speaker who had achieved Level 2-A (Grade A) in the Mandarin Proficiency Test. The machine voice was generated using the speech synthesis function (intellectual female voice model) of Wancai Animation Master software. To ensure the comparability of speech variables under different conditions, both types of voices were uniformly adjusted in terms of speaking rate (approximately 240 characters per minute), pitch, and pause structure, thereby minimizing interference from non-experimental variables.

#### 2.3.2 Manipulation check

To verify the effectiveness of the voice manipulation, a pilot test was conducted with 15 participants who did not take part in the main experiment. Participants evaluated the naturalness of the two voice types. Naturalness is a core subjective dimension in speech synthesis research and is commonly used to assess whether different voice conditions can be perceptually distinguished. It refers to the extent to which a voice sounds like natural human speech (Taylor, 2009). In this study, naturalness was measured using the Mean Opinion Score (MOS) on a five-point Likert scale (1 = very unnatural, 5 = very natural), a standardized method recommended by the International Telecommunication Union (ITU-T P.800). The results showed that the human voice (*M* = 4.73, SD = 0.46) was rated significantly more natural than the machine-generated voice (*M* = 2.13, SD = 0.64), *t*(14) = 15.92, *p* < 0.001, confirming a clear perceptual distinction between the two conditions and indicating a successful manipulation.

To ensure consistency in material difficulty across different experimental conditions, this study invited five graduate students in educational technology to evaluate the difficulty of the four video versions after production, using a five-point Likert scale (1 = very easy, 5 = very difficult). The results of the one-way repeated-measures analysis of variance showed that there was no significant difference in the difficulty of the four videos, *F*(3,12) = 2.216, *p* = 0.139, $\eta_{p}^{2}\;$ = 0.357. This indicates that the difficulty of the learning materials across experimental conditions was well comparable.

In addition, to mitigate potential confounding effects associated with the within-subject design, several control measures were implemented. First, all video materials were derived from the same course to ensure structural consistency. Second, a Latin square design was employed to counterbalance the presentation order of the four videos, thereby controlling for order and practice effects. Third, a 10-s fixation period and a 30-s rest interval were inserted between experimental blocks to allow participants to recover attentional states and return to baseline hemodynamic levels, thus reducing fatigue effects.

Each video lasted approximately 2 min. This duration was selected to achieve a balance between fNIRS signal quality and task effectiveness. It is sufficient to elicit stable hemodynamic responses while minimizing motion artifacts and attentional decline associated with longer tasks. Moreover, prior multimedia learning research has demonstrated that this duration is adequate for learners to achieve initial comprehension and integration of conceptual content, which is consistent with established fNIRS-based multimedia learning paradigms (e.g., Li et al., 2022).

#### 2.3.3 Prior knowledge questionnaire

The prior knowledge questionnaire was used to control for the influence of prior knowledge on the experimental results. The questionnaire consisted of two parts. The first part used a five-point Likert scale to assess participants’ subjective understanding of the pedagogical agent; the second part consisted of a seven-item objective test on aviation knowledge. There was no overlap in content between the prior knowledge test and the learning assessment. Data analysis revealed that the average score on the prior knowledge test was 2.37 (SD = 1.07, out of 7), indicating a generally low level of knowledge. No participant scored above 5, suggesting that participants had limited understanding of aviation knowledge.

#### 2.3.4 Learning outcome tests

The learning achievement test was administered after each instructional video to assess learning outcomes. Each video included 5 multiple-choice questions, 4 of which were retention tests to assess memory of the video content, and 1 was a transfer test to assess understanding and application of the knowledge. Each question was worth 5 points; with 4 videos totaling 20 questions, the maximum score was 100 points. All questions were adapted from the original question bank of the China University MOOC course.

### 2.4 Experimental procedure

**Figure 3:**
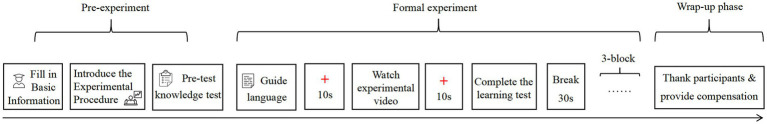
Experimental flowchart. “+” represents the “resting gaze point” stage (the purpose of which is to return the blood oxygen level in the subject’s brain to baseline). “...” represents “repeated learning and testing sessions”.

The experimental protocol was programmed using E-Prime 3.0 software. The stimulus display was a 23-inch monitor with a refresh rate of 64 Hz and a resolution of 1,280 × 768 pixels. The material was presented in blocks, with each block consisting of a video of a specific condition type and its corresponding test questions. After each stimulus, a red fixation mark (“+”) appeared in the center of the screen for 10 s. There were a total of four blocks. The block order was balanced across participants. Before proceeding to the next video learning session, participants were given a 30-s rest period to allow cerebral blood oxygen levels to return to baseline. The experimental procedure is shown in Figure 3.

The experiment was conducted in a fNIRS laboratory, with a total duration of approximately 30 min. The experiment consisted of three phases, a pre-test, a formal experiment, and a debriefing phase. In the pre-test phase, participants completed demographic information and the prior knowledge questionnaire. In the formal experiment phase, participants were first introduced to the experimental procedure and then fitted with the fNIRS device. Subsequently, participants watched four instructional videos, and after each video, they immediately completed the corresponding learning performance test. After all videos and tests were completed, the fNIRS equipment was removed by the experimenter, and the experiment concluded.

### 2.5 fNIRS data collection

**Figure 4:**
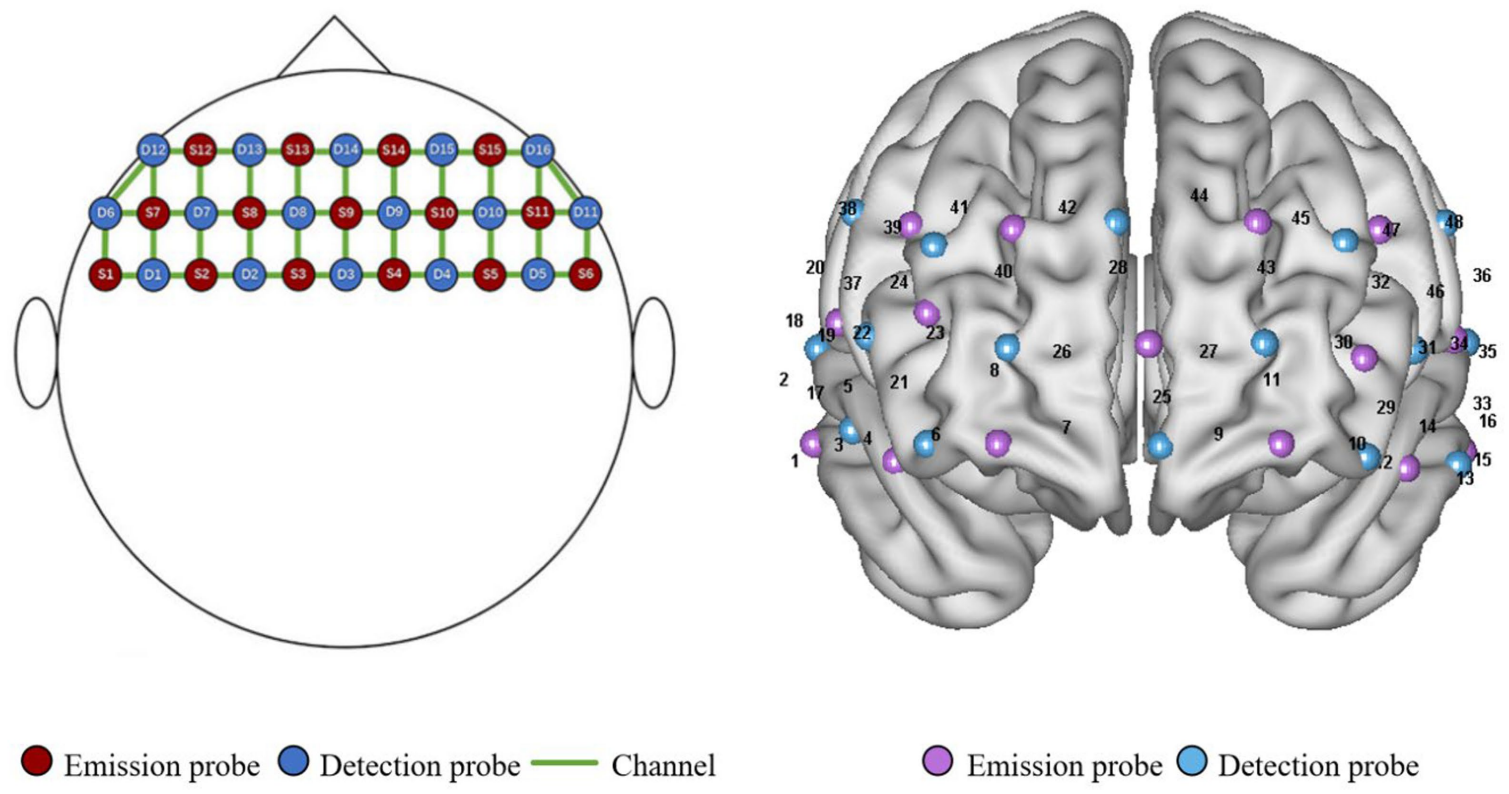
Schematic diagram of fNIRS channel arrangement.

This experiment used a portable near-infrared brain functional imaging system (NirSmart, Danyang Huichuang) to collect cerebral cortical oxygenation signals. The system utilizes near-infrared light at two wavelengths, 730 nm and 850 nm, with a sampling frequency of 11 Hz, to record in real time the relative changes in the concentrations of HbO, HbR, and total hemoglobin (HbT) in the prefrontal cortex while the subject performs the experiment. This instrument operates on the principle of three-dimensional localization and employs the internationally recognized 10/20 electrode placement system to achieve spatial localization. It then determines the brain regions associated with each channel based on Brodmann areas and the Automatic Anatomical Labeling (AAL) template. The device comprises a total of 15 emitter probes and 16 detector probes, spaced 3 centimeters apart. Each pair of adjacent light sources forms a channel, resulting in a total of 48 channels. The channel layout is shown in Figure 4. The probe configuration covers key brain regions such as the prefrontal, temporal, and parietal lobes, which are known to play crucial roles in various higher-order cognitive functions, including working memory, semantic processing, and social interaction (Csipo et al., 2021). This study primarily analyzes changes in the concentration of oxygenated hemoglobin. This metric typically rises significantly during neuronal activation, is sensitive to task responses, and has a high signal-to-noise ratio, making it a commonly used and effective indicator in fNIRS research (Pinti et al., 2020; Chen et al., 2022).

### 2.6 Data analysis procedure

#### 2.6.1 fNIRS data analysis

The NIR data were preprocessed using the NIRSpark software (Chen et al., 2025; Ma et al., 2025), following these steps: (1) Excluded time periods unrelated to the experimental task and conducted a preliminary inspection of all 48 channels in each dataset. Channels exhibiting persistent signal saturation, extremely low light intensity, or excessive noise were discarded. If the number of bad channels for a given participant exceeded 30% of the total number of channels, all of that participant’s data were discarded entirely (Brigadoi et al., 2014; Wang et al., 2025); (2) Spline interpolation was used to correct motion artifacts caused by head movement, thereby improving signal stability; (3) Apply a 0.01–0.2 Hz band-pass filter to remove physiological noise, including head motion, heartbeat, respiration, and low-frequency signal drift; (4) Convert the raw light intensity data into hemoglobin concentration values using the modified Beer–Lambert law. This study focuses primarily on changes in HbO concentration, as HbO is more sensitive to task-induced changes in cerebral blood flow (Hoshi et al., 2001; Li et al., 2024); (5) Each trial’s learning phase was defined as the task period, and a short pre-task resting interval immediately preceding each trial was selected as the baseline. As fNIRS signals reflect changes in hemoglobin concentration relative to a baseline period, a pre-stimulus time window is typically used as a reference state for estimating task-related brain activation (Pinti et al., 2020). On this basis, the experimental design function was convolved with the hemodynamic response function (HRF) to construct the design matrix. A general linear model (GLM) was then applied to estimate *β* values for each channel, representing the magnitude of task-related activation relative to baseline. For HbO signals, positive *β* values indicate task-related cortical activation enhancement, while negative *β* values indicate activation attenuation (Tian et al., 2021). Finally, a repeated-measures analysis of variance (ANOVA) was performed on the β values across different conditions. For *p*-value comparisons across channels, the False Discovery Rate (FDR) method was used for multiple comparison correction (*p* < 0.05); corrected *p*-values < 0.05 were considered statistically significant, thereby further reducing the false positive rate (Noble, 2009).

#### 2.6.2 Behavioral data analysis

Behavioral data and preprocessed NIR data were analyzed using SPSS 27.0 statistical analysis software. The significance level was set at 0.05. Descriptive statistics are reported as mean ± standard deviation. ANOVA was conducted on the accuracy (ACC) and response time (RT) of the participants’ learning performance (retention test and transfer test) to examine whether there were significant differences in learning outcomes across the four different conditions. Bonferroni *post hoc* multiple comparison corrections were applied to significant channels (*p* < 0.05), and simple effects analyses were further conducted for channels showing significant interaction effects.

## 3 Research result

### 3.1 Behavioral experiment results

ANOVA was conducted to analyze participants’ accuracy on the learning test and their behavioral reaction times; the descriptive statistics are presented in Table 1. The results showed that, on the retention test, neither the main effects of avatar type nor voice type were significant; however, the interaction between the two was significant [*F*(1, 29) = 12.083, *p* = 0.002, $\eta_{p}^{2}\;$ = 0.294]. Further simple effects analysis revealed that, under the animated agent condition, retention test scores for the human voice type (*M* = 14.83, SD = 4.639) were significantly higher than those for the machine voice type (*M* = 11.33, SD = 4.536); under the human avatar condition, there was no significant difference in retention test scores based on voice type, *F*(1, 29) = 3.511, *p* = 0.071, $\eta_{p}^{2}\;$ = 0.108. Under the human voice condition, the retention test scores for the animated avatar (*M* = 14.83, SD = 4.639) were significantly higher than those for the human avatar (*M* = 12.83, SD = 3.640); Under the machine voice condition, the retention test scores for the human avatar (*M* = 14.33, SD = 3.880) were significantly higher than those for the animated avatar (*M* = 11.33, SD = 4.536). These results indicate a clear interaction between the visual appearance and voice type of instructional agents in retention tests, meaning that the performance of instructional agents with different visual appearances under different voice conditions is not independent but mutually influences one another. Animated agents paired with human voices achieved the best retention scores, while those paired with machine voices performed the worst; the compatibility between visual appearance and voice is crucial for the retention of basic knowledge.

**Table 1:** Descriptive statistics of learning performance on instructional videos (M ± SD).

**Variable**	**Human agent**				**Animated agent**			
	**Human voice**		**Machine voice**		**Human voice**		**Machine voice**	
	***M***	***SD***	***M***	***SD***	***M***	***SD***	***M***	***SD***
Retention scores	12.83	3.640	14.33	3.880	14.83	4.639	11.33	4.536
Transfer scores	2.83	2.520	1.50	2.330	3.33	2.397	1.33	2.249
Response time	6582.08	1072.908	5892.58	1188.706	6561.42	1165.914	6148.10	1117.121

**Figure 5:**
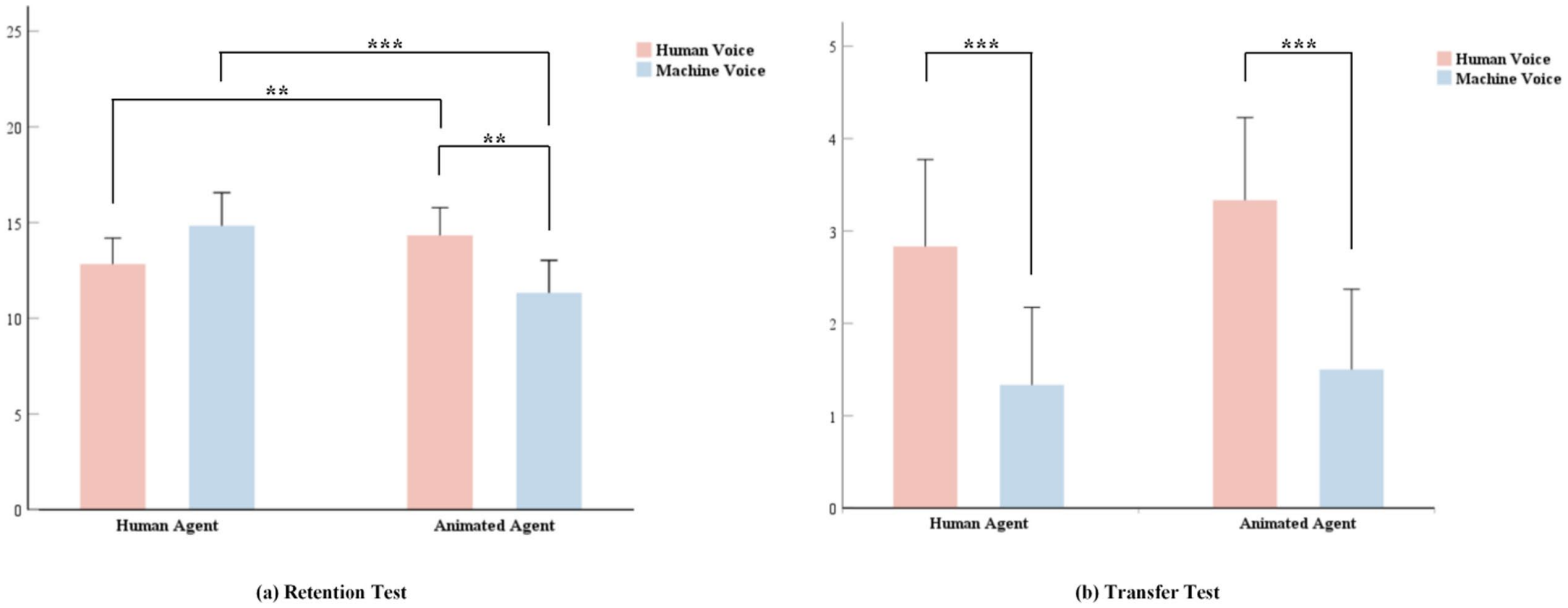
Interaction between pedagogical agent image and voice type on test scores. **p < 0.01; ***p < 0.001.

For the transfer test, the main effect of voice type was significant, *F*(1, 29) = 14.500, *p* < 0.001, η_p_^2^ = 0.333, with transfer test scores for the human voice (*M* = 3.083, SD = 0.332) being significantly higher than those for the machine voice (*M* = 1.417, SD = 0.286). The main effect of image type was not significant, *F*(1, 29) = 0.162, *p* = 0.690, $\eta_{p}^{2}\;$ = 0.006, and the interaction was also not significant, *F*(1, 29) = 0.563, *p* = 0.459, $\eta_{p}^{2}\;$ = 0.019. These results indicate that voice type is a key factor influencing knowledge transfer. Compared with synthetic machine voices, human voices, with their natural prosody and emotional variation, more effectively promote knowledge transfer. The interaction between pedagogical agent image and voice type on test scores is shown in Figure 5.

ANOVA on response time revealed a significant main effect of voice type, *F*(1, 29) = 10.175, *p* = 0.003, $\eta_{p}^{2}\;$ = 0.260, with response time for the human voice (*M* = 6571.752, SD = 183.265) being significantly longer than that for the machine voice (*M* = 6020.340, SD = 181.574). The main effect of image type and the interaction were not significant. Combining the results of the transfer test reveals that real human voices were associated with longer reaction times but also higher performance levels. This finding may indicate that learners engaged in more cognitive processing or demonstrated greater task engagement under these conditions.

### 3.2 fNIRS results

**Figure 6:**
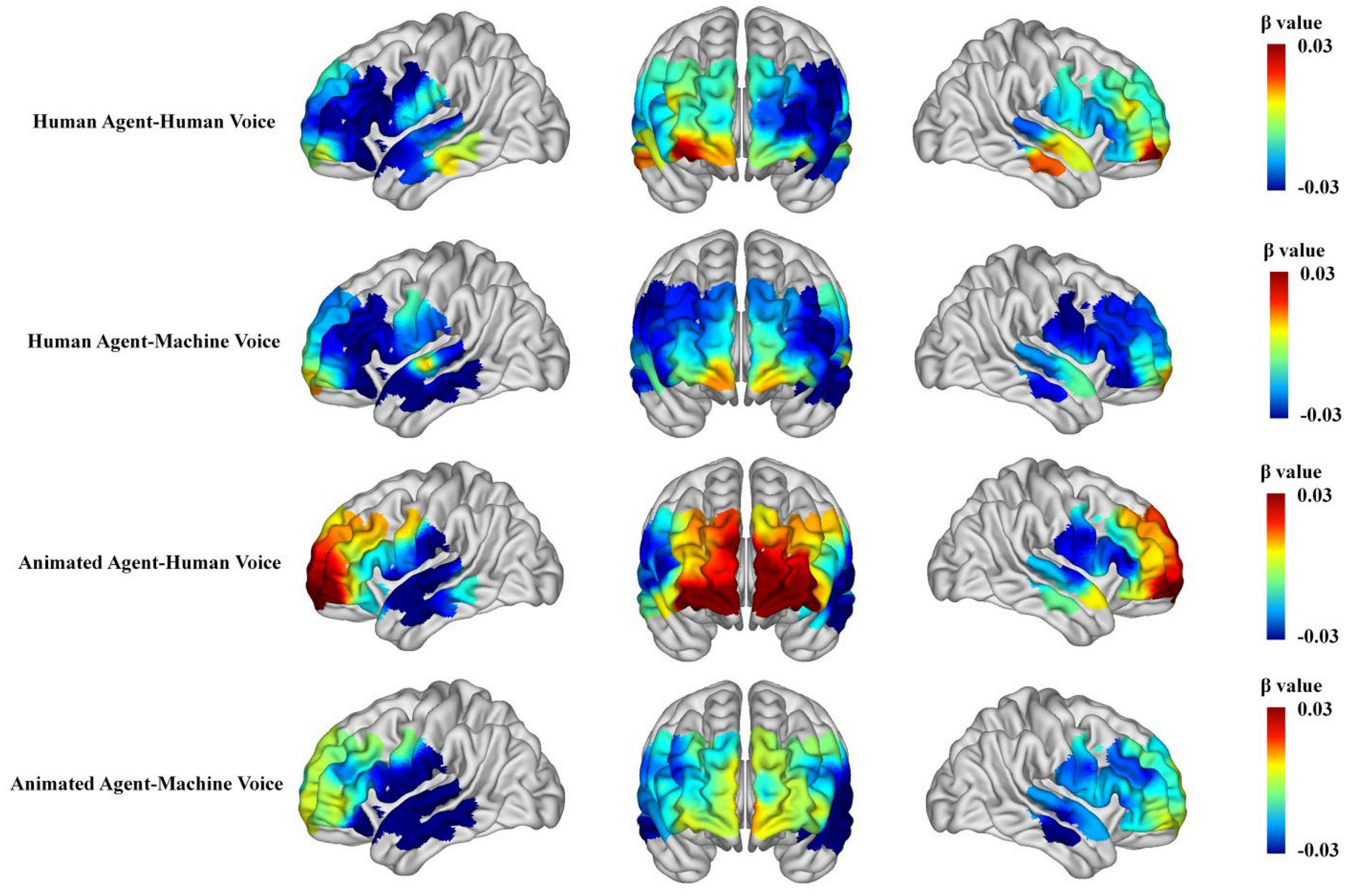
Comparison of brain region activation across the four conditions.

A channel-wise repeated-measures ANOVA was conducted using SPSS, with a 2 (image type: human agent, animated agent) × 2 (voice type: human voice, machine voice) design. The FDR method was applied across channels to correct *p*-values for multiple comparisons, further reducing the false positive rate. The results showed that the main effect of image type was significant in the inferior frontal gyrus (CH29), middle temporal gyrus (CH33), frontal pole regions (CH28, CH30, CH40, CH43), and dorsolateral prefrontal cortex (CH45, CH47), indicating that brain activation intensity induced by the animated agent was significantly higher than that induced by the human agent. The main effect of voice type was significant in the frontal pole region (CH06), middle temporal gyrus (CH15, CH16), and dorsolateral prefrontal cortex (CH24), indicating that brain activation intensity induced by the human voice was significantly higher than that induced by the machine voice. The interaction between image and voice was significant in prefrontal cortex channel CH27, *F*(1, 29) = 7.765, *p* = 0.009, $\eta_{p}^{2}$ = 0.217. Simple effects analysis revealed that, under the animated agent condition, brain activation intensity induced by the human voice was significantly higher than that induced by the machine voice, *F*(1, 29) = 6.560, *p* = 0.016, $\eta_{p}^{2}$ = 0.190; under the human agent condition, there was no significant difference in activation intensity between the two voice types. Under the human voice condition, brain activation intensity induced by the animated agent was significantly higher than that induced by the human agent, *F*(1, 29) = 8.235, *p* = 0.008, $\eta_{p}^{2}\;$ = 0.227; under the machine voice condition, there was no significant difference in activation intensity between the two image types. These findings indicate that the combination of an animated agent and a human voice elicited the strongest brain activation, primarily in the frontal pole, dorsolateral prefrontal cortex, and middle temporal gyrus. The interaction effect was concentrated in prefrontal channel CH27, suggesting that this region is particularly sensitive to multimodal social cue integration, while other brain regions mainly showed independent main effects of image or voice, indicating that the two factors may play relatively independent modulatory roles in different brain regions. Detailed information on significantly activated channels (*p* < 0.05) is presented in Table 2. Differences in brain activation during learning across the four conditions are shown in Figure 6.

**Table 2:** Significant results of the analysis of variance (ANOVA) across different conditions.

**Effect**	**Channel**	**Region (Brodmann areas)**	***F*(1,29)**	***p***	$\eta_{p}^{2}$
Main effect of image type	CH28	10-Frontopolar area	4.688	0.039	0.139
	CH29	47-Inferior prefrontal gyrus	6.000	0.021	0.171
	CH30	10-Frontopolar area	9.012	0.005	0.237
	CH33	21-Middle temporal gyrus	6.076	0.020	0.173
	CH40	10-Frontopolar area	4.561	0.041	0.136
	CH43	10-Frontopolar area	7.553	0.010	0.207
	CH45	9-Dorsolateral prefrontal cortex	4.987	0.033	0.147
	CH47	9-Dorsolateral prefrontal cortex	6.963	0.013	0.194
Main effect of voice type	CH06	10-Frontopolar area	4.187	0.050	0.126
	CH15	21-Middle temporal gyrus	5.211	0.030	0.152
	CH16	21-Middle temporal gyrus	4.874	0.035	0.144
	CH24	46-Dorsolateral prefrontal cortex	6.674	0.015	0.192
Image type × Voice type interaction	CH27	10-Frontopolar area	7.765	0.009	0.217

*The *p* was corrected using FDR method across fNIRS channels.

### 3.3 Relationship between fNIRS data and behavioral data

To explore the intrinsic relationship between brain activation and behavioral performance, Pearson correlation analyses were conducted between the *β*-values of activation intensity in channels showing significant main effects and test scores (retention scores, transfer scores, and total scores). Selected results are presented in Table 3. The results showed that significant correlations were observed only in the human agent with human voice condition. Activation levels in the frontal pole channel CH06 were significantly positively correlated with retention scores (*r* = 0.385, *p* = 0.036), and activation levels in the middle temporal gyrus channel CH15 were significantly positively correlated with both retention scores (*r* = 0.392, *p* = 0.032) and total scores (*r* = 0.406, *p* = 0.026). Under the remaining conditions, no significant correlations were found between brain region activation and learning performance (*p* > 0.05), and transfer scores were not significantly correlated with brain activation indicators under any condition. These results indicate that under the condition where a human image is matched with human voice, learners’ learning performance is stably associated with activation in prefrontal and temporal lobe regions. Specifically, learners with higher retention scores exhibited stronger activation in the frontal pole and middle temporal gyrus, which may reflect greater cognitive resource investment and more effective information encoding and integration processing during learning.

**Table 3:** Selected results of Pearson correlation analysis between test performance and fNIRS data.

**Channel**	**Variable**	**Human agent**				**Animated agent**			
		**Human voice**		**Machine voice**		**Human voice**		**Machine voice**	
		***r***	***p***	***r***	***p***	***r***	***p***	***r***	***p***
CH06	Retention scores	0.385	0.036*	−0.065	0.732	−0.023	0.905	0.012	0.950
	Transfer scores	−0.156	0.411	0.150	0.430	−0.340	0.066	−0.125	0.511
	Total scores	0.244	0.193	−0.046	0.811	−0.168	0.376	−0.042	0.825
CH15	Retention scores	0.392	0.032*	0.012	0.948	−0.238	0.206	0.110	0.563
	Transfer scores	0.195	0.302	0.040	0.833	−0.113	0.554	0.239	0.203
	Total scores	0.406	0.026*	−0.036	0.851	−0.025	0.183	0.193	0.307

*p < 0.05; The *p* was corrected using FDR method across fNIRS channels.

## 4 Discussion

Based on social agency theory and cognitive load theory, this study employed fNIRS technology to investigate the effects of pedagogical agent image type and voice type on learning performance and brain activation patterns. The results revealed that the visual and auditory cues of pedagogical agents jointly influenced the learning process in an interactive manner, with effects observed not only in behavioral outcomes but also in activation patterns of the prefrontal and temporal brain regions. These findings suggest that the effectiveness of pedagogical agent design depends not merely on individual cues but rather on the synergistic integration of multimodal social cues during learning.

### 4.1 Effects of pedagogical agent image, voice, and their interaction on learning performance

The behavioral results of this study showed that the main effect of pedagogical agent image type was not significant, indicating that there was no significant difference in overall learning outcomes between animated pedagogical agents and human pedagogical agents. Although this finding is inconsistent with some earlier studies, it aligns with recent research. For example, Polat et al. (2025) found that human instructors and humanlike animated pedagogical agents in video learning showed no significant differences in learning outcomes, engagement, or social presence. Lawson and Mayer (2022) compared human and humanlike animated pedagogical agents and also found no significant difference in learners’ learning performance. Zhao F. Z. et al. (2025) further pointed out that although students reported higher social connection ratings for human teachers compared to animated pedagogical agents, there was no significant difference in learning outcomes between the two. This finding supports the media equivalence hypothesis, which posits that learners unconsciously treat media as social actors during interaction, applying social interaction rules from real human-human interactions to human-computer interaction contexts (Lawson and Mayer, 2022). Therefore, regardless of whether the pedagogical agent image is human or animated, learners interact with it using similar social cognitive patterns, resulting in non-significant differences in learning performance (Zhao F. Z. et al., 2025).

Second, the main effect of voice type was significant, with the human voice yielding significantly better transfer performance than the machine voice, accompanied by longer response times. From the perspective of social agency theory, the rich intonation, rhythm, and emotional variation inherent in human voices better stimulate learners’ social presence, thereby promoting deeper understanding and transfer application of knowledge (Fiorella and Mayer, 2022; Atkinson et al., 2005). Moreover, human voices resulted in longer response times but corresponded to better transfer performance, suggesting that they may have facilitated a higher degree of cognitive engagement or processing depth. This result supports cognitive load theory, which holds that moderately increased processing investment facilitates meaning construction and knowledge integration (Sweller, 2020). It should be noted that the transfer test in this study included only one item per condition. While this can provide a preliminary indication of learners’ ability to transfer knowledge, it has certain limitations in terms of measurement reliability. Therefore, the above conclusions should be regarded as exploratory findings and await validation in future studies using multi-item measures.

Finally, in the retention test, a significant interaction effect between pedagogical agent image and voice was observed, indicating that learners’ processing of pedagogical agents depends on the integration of multimodal social cues. This finding further supports social agency theory, which suggests that social cues such as voice and image can trigger learners’ social responses, promote human–computer interaction, drive deeper cognitive processing, and thereby lead to meaningful learning outcomes (Mayer et al., 2003; Mayer and DaPra, 2012; Fiorella and Mayer, 2022). Specifically, the animated agent performed best under the human voice condition and worst under the machine voice condition. This suggests that when the visual channel authenticity is low, natural and authentic human voices play a compensatory role in maintaining social presence and supporting cognitive processing. Conversely, machine voices, lacking the emotional and interactive properties of human speech, struggle to support the integration of social cues from the animated agent, potentially leading to inefficient allocation of cognitive resources and thus reduced learning outcomes. These results partially support Hypothesis 1, Hypothesis 2 and Hypothesis 3.

### 4.2 Effects of pedagogical agent image and voice on brain activation patterns

The fNIRS results provided clear neurophysiological evidence for the behavioral findings. The results showed that both the visual image and voice type of pedagogical agents significantly activated the prefrontal–temporal network associated with semantic processing, working memory, executive control, and social cognition, indicating that learners simultaneously engaged in content comprehension and social cue integration when processing pedagogical agent information. Existing fNIRS studies on pedagogical agents have also shown that embodied pedagogical agents enhance learning performance and elicit stronger activity in social-related brain regions (Yin et al., 2025), which is consistent with the predictions of social agency theory.

Regarding image type, the animated agent induced stronger brain activation than the human agent in the inferior frontal gyrus, middle temporal gyrus, frontal pole regions, and dorsolateral prefrontal cortex. Previous research has shown that the prefrontal cortex, especially the frontal pole and dorsolateral prefrontal cortex, is typically associated with higher-order cognitive control, working memory updating, and metacognitive monitoring (Pinti et al., 2020). The middle temporal gyrus is closely related to semantic representation, semantic retrieval, and event comprehension (Davey et al., 2016), while the inferior frontal gyrus is involved in semantic selection, inhibition of competing information, and controlled retrieval (Grindrod et al., 2008). These results support Hypothesis 4, suggesting that animated agents may prompt learners to invest more cognitive resources in social cue integration and information processing, thereby activating the associated higher-order cognitive brain regions. Regarding voice type, the human voice induced stronger activation than the machine voice in the frontal pole, these results support Hypothesis 5, middle temporal gyrus, and dorsolateral prefrontal cortex. This indicates that the natural prosody, rhythm, and emotional cues of the human voice more readily trigger learners’ cognitive processing and social perception, thereby enhancing activity levels in brain regions related to semantic processing and executive control. Further analysis revealed a significant interaction between the agent’s image and voice in the prefrontal cortex, with the combination of an animated agent and a human voice eliciting the strongest brain activation, this result supports Hypothesis 6. This study provide neurobiological support for the CASTLE model, when multiple social cues are presented simultaneously, they synergistically activate social schemas in the learner’s long-term memory, thereby amplifying the facilitative effects on cognitive and emotional processes (Schneider et al., 2022). This suggests that learners do not process individual cues in isolation, but rather integrate visual and auditory information into a unified social representation.

This study found that different multimodal combinations exhibit distinct characteristics in terms of learning outcomes and neural mechanisms. On the one hand, with the combination of human voice and human agent, both visual and auditory cues are highly realistic and consistent in source, this consistency helps learners more easily integrate multisensory social cues, thereby activating existing social cognitive schemas (Schneider et al., 2022; Mayer, 2020). Prior research has shown that when multimedia information conveys consistent social cues across different channels, learners tend to experience lower cognitive load and are more likely to engage in automatic semantic integration processes (Mayer and Moreno, 2003). In this case, activation in fronto-temporal regions may be positively associated with learning performance, potentially reflecting efficient task-related cognitive engagement. Moreover, fNIRS has been demonstrated to effectively capture differences in cognitive load and information integration during multimedia learning, with activation in the prefrontal cortex—particularly the frontopolar cortex and dorsolateral prefrontal cortex—being closely related to learning efficiency (Ma et al., 2025). On the other hand, in the animated agent–human voice condition, the visual and auditory social cues differ in terms of perceived realism. Prior multimedia learning research suggests that when information across channels is socially incongruent, learners need additional cross-modal integration processes to construct a unified mental representation (Fiorella and Mayer, 2022). This process may require greater engagement of executive control resources, particularly in regions such as the dorsolateral prefrontal cortex and frontopolar cortex, which are associated with cognitive control and information integration (Funahashi, 2022). Therefore, the increased overall brain activation observed in this condition may reflect a compensatory processing mechanism, whereby the brain recruits additional neural resources to maintain cognitive performance under higher integration demands (Varnosfaderani et al., 2024). Previous research has shown that there is not necessarily a simple linear relationship between brain activation levels and task performance; rather, this relationship is influenced by factors such as task difficulty, the allocation of cognitive resources, and processing efficiency (Poldrack, 2006; Di Domenico et al., 2015). So, higher brain activation in this study is more likely to reflect more complex information integration and executive control processes, rather than directly indicating better or worse learning performance.

In summary, the image and voice of the pedagogical agent influence learners’ semantic processing, cognitive control, and social information integration by modulating the coordinated activity of the prefrontal-temporal network, thereby collectively contributing to learning outcomes. Therefore, the design of pedagogical agents should not only consider the merits of individual cues but also focus on the relationships between them. Optimal learning outcomes can arise from two distinct pathways: first, efficient neural processing under highly consistent cues; and second, deep cognitive integration triggered by complementary cues. This finding offers a new theoretical perspective for understanding the mechanisms underlying the learning facilitation of multimodal social cues and provides a neurobiological basis for the design of adaptive pedagogical agents.

### 4.3 Implications and limitations

This study investigated the effects of pedagogical agent image and voice on learning at both behavioral and neural mechanism levels, providing behavioral and neurophysiological evidence for social agency theory and the media equivalence hypothesis. The practical implication of this study is that, rather than pursuing high realism in a single modality, greater attention should be paid to the coordination and matching of social cues conveyed by pedagogical agents’ visual and auditory channels. The combination of an animated agent with a human voice yielded the most optimal learning outcomes at both behavioral and neural levels. This may be because the lower visual realism of the animated agent reduces attention diversion unrelated to the learning content, whereas the human voice provides rich prosodic and emotional cues that effectively elicit learners’ social responses. Therefore, instructional design of pedagogical agents should focus on the integrative optimization of multimodal cues, designing agents with human-like characteristics that provide meaningful social signals, thereby activating learners’ social schemas, guiding attention toward learning content, and ultimately supporting learning.

Although this study provides several evidence-based implications for both theory and practice, it still has certain limitations. First, regarding the design of pedagogical agent image, although key variables such as agent position, background, size, and expressive style were controlled, there may still be other visual social cues that were not fully accounted for, such as the visual attractiveness of the agent. Prior research has shown that visual attractiveness can influence learners’ attention allocation and cognitive processing and may independently affect learning outcomes (Domagk, 2010; Zhao Y. M. et al., 2025). In addition, emotional valence (positive vs. negative), gestures, and other agent characteristics (e.g., age and role) may also influence learning performance (Davis et al., 2022; Wang et al., 2021; Makransky et al., 2019). Furthermore, the combination of human agent and machine voice is relatively uncommon in real instructional settings, which may pose certain limitations to ecological validity. Although this design helps isolate the effects of different social cues and enhances internal validity, its practical applicability in real-world learning contexts still requires further validation. Therefore, future research should balance experimental control with more ecologically valid designs to improve the external generalizability of the findings.

Second, this study primarily focused on how the pedagogical agent’s image and voice influence learners’ learning performance and neural activation patterns. However, it did not include learners’ subjective perceptions of the agent itself, such as liking, trust, or perceived social presence. Prior research has shown that learners’ subjective perceptions of pedagogical agents can substantially influence their motivation and cognitive engagement, and may play a mediating or moderating role in multimedia learning outcomes (Edwards et al., 2019). Therefore, future studies should incorporate measures of learners’ subjective perceptions of pedagogical agents into the experimental design.

Third, the learning materials used in this study were aerospace-related content, and the video duration was relatively limited. Previous research has indicated that both the domain of learning content and the structure of instructional materials can significantly influence multimedia learning outcomes (Polat, 2023; Guo et al., 2014). Therefore, the generalizability of the findings to different subject domains and longer learning tasks remains to be further examined. In addition, the sample consisted mainly of university students with a relatively higher proportion of female participants, which may limit the generalizability of the findings to more gender-balanced populations (Xiao et al., 2025). Future research should therefore validate the findings in more diverse populations to enhance external validity.

Finally, this study adopted a within-subjects design. Although this design helps control for individual differences and improves statistical power, it is also more susceptible to order effects, practice effects, and fatigue effects (Charness et al., 2012). While the present study attempted to control for these issues through counterbalancing of presentation order, future studies may further address potential order effects by adopting a Latin square design, conducting additional statistical tests on order as a factor, or using between-subjects designs to more thoroughly rule out potential confounds.

## 5 Conclusion

The findings revealed that: (1) The voice type of pedagogical agents significantly influenced transfer test performance, with human voices promoting learning outcomes more effectively than machine voices. (2) The image and voice information of pedagogical agents shared common brain regions in the prefrontal cortex, middle temporal gyrus, and dorsolateral prefrontal cortex, while the brain region specific to image type was located in the inferior frontal gyrus. (3) Correlation analysis revealed that only when the pedagogical agents was a combination of human voice and human agent did activation levels in the prefrontal-temporal brain regions show a significant positive correlation with academic performance. (4) The combination of an animated agent with a human voice yielded the best learning performance and corresponded to stronger levels of brain activation. This study provides evidence at the cognitive neural level that the presentation of pedagogical agents can facilitate learners’ cognitive processing and offers practical implications for optimizing multimedia instructional videos.

## Data Availability

The raw data supporting the conclusions of this article will be made available by the authors, without undue reservation.
