# Supplementary material for: Neural mechanisms of video learning influenced by pedagogical agents’ image and voice: an fNIRS study
Source: Front Psychol. 2026 Jul 10;17:1863163. doi: 10.3389/fpsyg.2026.1863163 (PMC13396010; doi:10.3389/fpsyg.2026.1863163)
Supplement: Supplementary file 1 [file Table_1.DOCX]

# Supplementary Material

**Table: Correspondence between spatial registration information of 48 NIRS channels in the experiment and Brodmann areas**

| **Channel** | **MNI Coordinates** | | | **Region (Brodmann areas)** | **Proportion** |
| --- | --- | --- | --- | --- | --- |
|  | **X** | **Y** | **Z** |  |  |
| CH1 | 71 | -12 | -6 | 21-Middle temporal gyrus | 0.98 |
| CH2 | 72 | -27 | 12 | 22-Superior temporal gyrus | 0.44 |
| CH3 | 59 | 18 | 1 | 38-Temporopolar area | 0.54 |
| CH4 | 54 | 45 | 1 | 47-Inferior prefrontal gyrus | 1 |
| CH5 | 60 | 29 | 16 | 47-Inferior prefrontal gyrus | 0.62 |
| CH6 | 39 | 64 | 6 | 10-Frontopolar area | 0.60 |
| CH7 | 15 | 74 | 8 | 11-Orbitofrontal area | 0.52 |
| CH8 | 27 | 65 | 25 | 10-Frontopolar area | 1 |
| CH9 | -15 | 73 | 7 | 11-Orbitofrontal area | 0.62 |
| CH10 | -40 | 61 | 1 | 11-Orbitofrontal area | 0.49 |
| CH11 | -29 | 63 | 22 | 10-Frontopolar area | 0.98 |
| CH12 | -54 | 40 | -3 | 38-Temporopolar area | 0.59 |
| CH13 | -62 | 5 | -9 | 21-Middle temporal gyrus | 0.99 |
| CH14 | -59 | 23 | 12 | 22-Superior temporal gyrus | 0.43 |
| CH15 | -71 | -19 | -10 | 21-Middle temporal gyrus | 0.93 |
| CH16 | -70 | -34 | 8 | 21-Middle temporal gyrus | 0.75 |
| CH17 | 68 | 1 | 16 | 22-Superior temporal gyrus | 0.63 |
| CH18 | 69 | -14 | 31 | 43-Subcentral area | 0.37 |
| CH19 | 63 | 11 | 31 | 44-Pars opercularis_part of Broca's area | 0.57 |
| CH20 | 64 | -9 | 43 | 6-Pre-motor and supplementary motor cortex | 0.74 |
| CH21 | 47 | 51 | 19 | 10-Frontopolar area | 0.47 |
| CH22 | 51 | 34 | 32 | 46-Dorsolateral prefrontal cortex | 0.77 |
| CH23 | 35 | 50 | 36 | 10-Frontopolar area | 0.93 |
| CH24 | 41 | 33 | 46 | 46-Dorsolateral prefrontal cortex | 0.77 |
| CH25 | -1 | 65 | 24 | 10-Frontopolar area | 0.87 |
| CH26 | 13 | 59 | 39 | 10-Frontopolar area | 1 |
| CH27 | -14 | 58 | 39 | 10-Frontopolar area | 1 |
| CH28 | -2 | 47 | 51 | 10-Frontopolar area | 0.99 |
| CH29 | -48 | 47 | 14 | 47-Inferior prefrontal gyrus | 0.59 |
| CH30 | -38 | 48 | 32 | 10-Frontopolar area | 0.91 |
| CH31 | -53 | 30 | 27 | 45-pars triangularis Broca's area | 0.65 |
| CH32 | -44 | 30 | 42 | 46-Dorsolateral prefrontal cortex | 0.87 |
| CH33 | -67 | -6 | 11 | 21-Middle temporal gyrus | 0.59 |
| CH34 | -64 | 5 | 26 | 22-Superior temporal gyrus | 0.51 |
| CH35 | -68 | -20 | 27 | 42-Primary and auditory association corte | 0.67 |
| CH36 | -65 | -14 | 38 | 43-Subcentral area | 0.46 |
| CH37 | 53 | 15 | 44 | 45-pars triangularis Broca’s area | 0.36 |
| CH38 | 54 | -5 | 56 | 9-Dorsolateral prefrontal cortex | 0.51 |
| CH39 | 44 | 16 | 57 | 9-Dorsolateral prefrontal cortex | 0.64 |
| CH40 | 21 | 44 | 51 | 10-Frontopolar area | 0.89 |
| CH41 | 27 | 27 | 59 | 9-Dorsolateral prefrontal cortex | 0.89 |
| CH42 | 11 | 34 | 61 | 9-Dorsolateral prefrontal cortex | 0.83 |
| CH43 | -25 | 43 | 48 | 10-Frontopolar area | 0.92 |
| CH44 | -14 | 33 | 60 | 9-Dorsolateral prefrontal cortex | 0.84 |
| CH45 | -33 | 25 | 56 | 9-Dorsolateral prefrontal cortex | 0.85 |
| CH46 | -56 | 11 | 37 | 45-pars triangularis Broca’s area | 0.41 |
| CH47 | -46 | 14 | 54 | 9-Dorsolateral prefrontal cortex | 0.61 |
| CH48 | -57 | -10 | 50 | 6-Pre-motor and supplementary motor cortex | 0.81 |
